# Supplementary material for: Genome-Wide Identification and Characterization of the HMGR Gene Family in Taraxacum kok-saghyz Provide Insights into Its Regulation in Response to Ethylene and Methyl Jsamonate Treatments
Source: Plants (Basel). 2024 Sep 21;13(18):2646. doi: 10.3390/plants13182646 (PMC11435204; doi:10.3390/plants13182646)
Supplement: Supplementary file 1 [file plants-13-02646-s001.zip › Figure S1. Prediction of transmembrane helices of TkHMGR1, TkHMGR2, TkHMGR6.pdf]

(A)

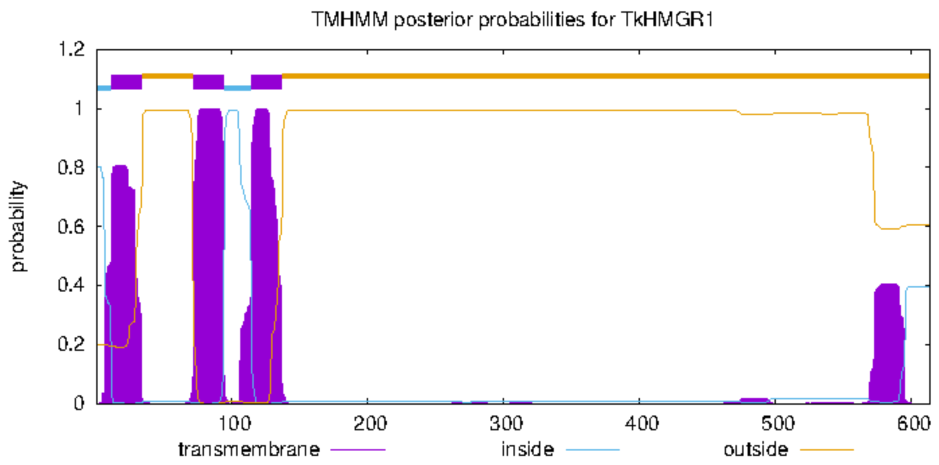

(B)

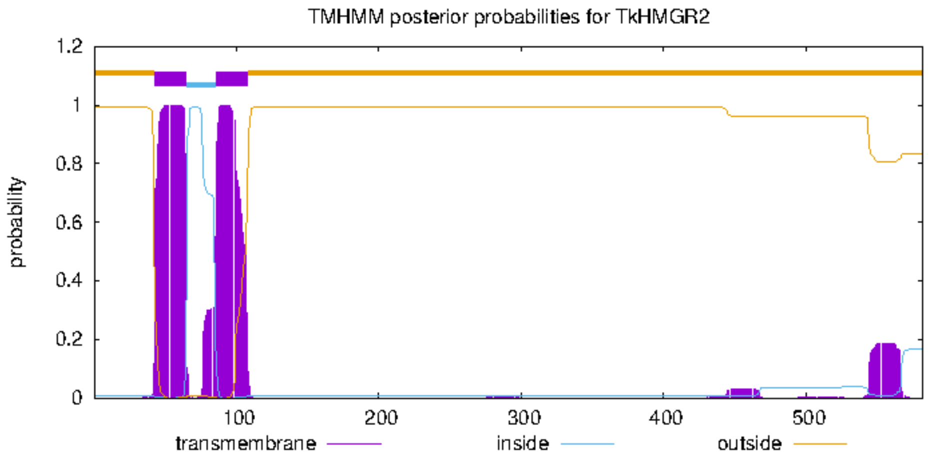

(C)

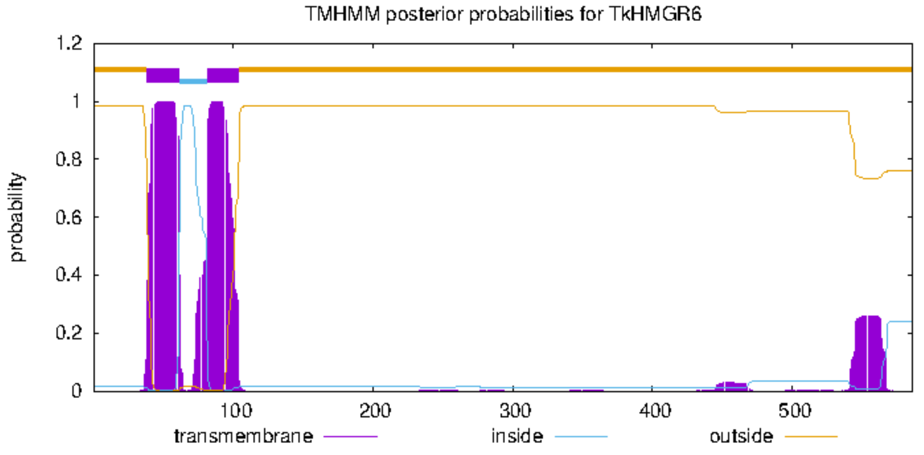

Figure S1. Prediction of transmembrane helices of TkHMGR1, TkHMGR2, TkHMGR6. (**A**) TkHMGR1 is predicted to have three transmembrane helices. (**B**) TkHMGR2 is predicted to have two transmembrane helices. (**C**) TkHMGR6 is predicted to have two transmembrane helices.
