# Supplementary material for: Genome-Wide Identification and Characterization of the HMGR Gene Family in Taraxacum kok-saghyz Provide Insights into Its Regulation in Response to Ethylene and Methyl Jsamonate Treatments
Source: Plants (Basel). 2024 Sep 21;13(18):2646. doi: 10.3390/plants13182646 (PMC11435204; doi:10.3390/plants13182646)
Supplement: Supplementary file 1 [file plants-13-02646-s001.zip › Figure S2. Results of protein three-dimensional structure prediction for TkHMGR proteins..pdf]

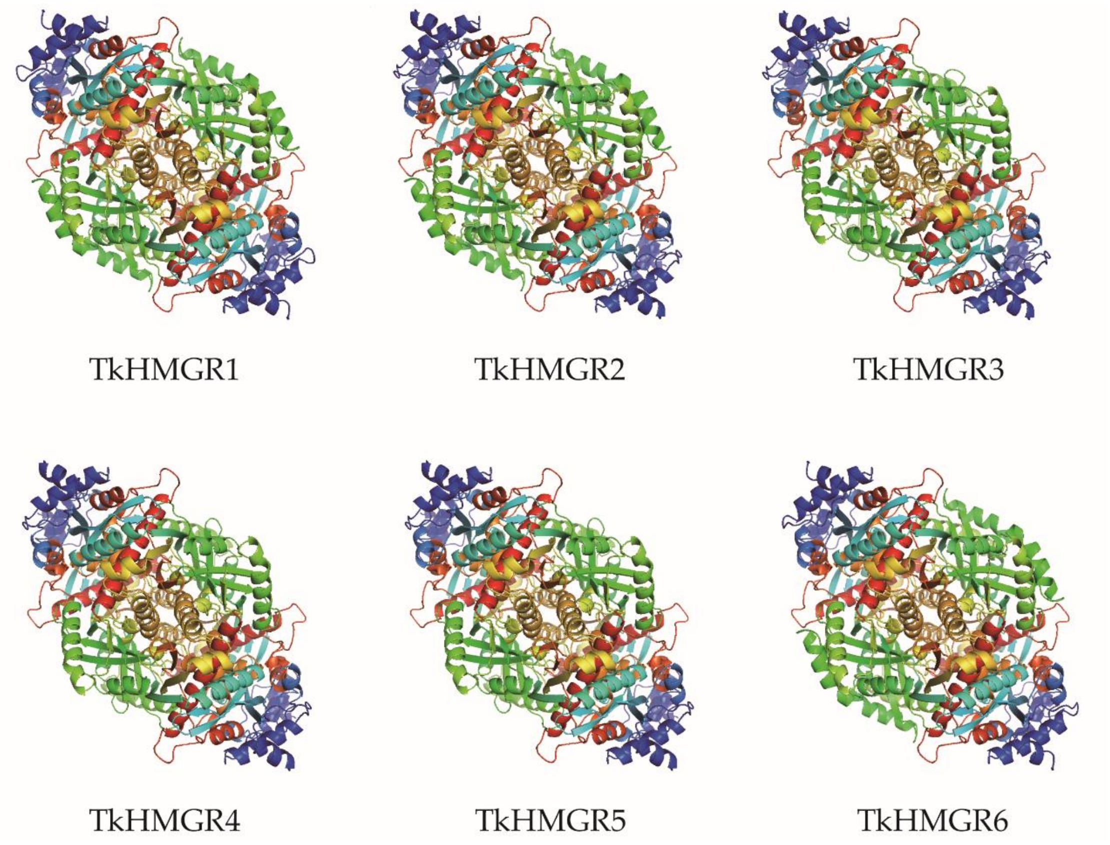

Figure S2. Results of protein three-dimensional structure prediction for TkHMGR proteins. TkHMGR1~TkHMGR6 have similar protein structures.
